# Infection or a third dose of mRNA vaccine elicits neutralizing antibody responses against SARS-CoV-2 in kidney transplant recipients

**DOI:** 10.1126/scitranslmed.abl6141

**Published:** 2022-02-01

**Authors:** Xavier Charmetant, Maxime Espi, Ilies Benotmane, Véronique Barateau, Francoise Heibel, Fanny Buron, Gabriela Gautier-Vargas, Marion Delafosse, Peggy Perrin, Alice Koenig, Noëlle Cognard, Charlène Levi, Floriane Gallais, Louis Manière, Paola Rossolillo, Eric Soulier, Florian Pierre, Anne Ovize, Emmanuel Morelon, Thierry Defrance, Samira Fafi-Kremer, Sophie Caillard, Olivier Thaunat

**Affiliations:** ^1^ CIRI, INSERM U1111, Université Claude Bernard Lyon I, CNRS UMR5308, Ecole Normale Supérieure de Lyon, Univ. Lyon, 21 avenue Tony Garnier, 69007 Lyon, France.; ^2^ Department of Nephrology and Transplantation, Strasbourg University Hospital, 67000 Strasbourg, France.; ^3^ Department of Virology, Strasbourg University Hospital, 67000 Strasbourg, France.; ^4^ Inserm UMR S1109, LabEx Transplantex, Fédération de Médecine Translationnelle de Strasbourg (FMTS), Université de Strasbourg, 67000 Strasbourg, France.; ^5^ Hospices Civils de Lyon, Edouard Herriot Hospital, Department of Transplantation, Nephrology and Clinical Immunology, 5, place d’Arsonval, 69003 Lyon, France.; ^6^ Claude Bernard University (Lyon 1), 43 boulevard du 11 Novembre 1918, 69622 Villeurbanne France.; ^7^ Institut de Génétique et de Biologie Moléculaire et Cellulaire (IGBMC), Centre National de la Recherche Scientifique (CNRS), UMR 7104, Institut National de la Santé et de la Recherche Médicale (INSERM), U1258, Université de Strasbourg, 67400 Illkirch, France.; ^8^ Eurofins Biomnis Laboratory, 69007 Lyon, France.

## Abstract

Transplant recipients, who receive therapeutic immunosuppression to prevent graft rejection, are characterized by high coronavirus disease 2019 (COVID-19)-related mortality and defective response to vaccines. We observed that previous infection with severe acute respiratory syndrome coronavirus 2 (SARS-CoV-2), but not the standard two-dose regimen of vaccination, provided protection against symptomatic COVID-19 in kidney transplant recipients. We therefore compared the cellular and humoral immune responses of these two groups of patients. Neutralizing anti-Receptor Binding Domain (RBD) IgG antibodies were identified as the primary correlate of protection for transplant recipients. Analysis of virus-specific B and T cell responses suggested that the generation of neutralizing anti-RBD IgG may have depended upon cognate T-B cell interactions that took place in germinal center, potentially acting as a limiting checkpoint. High dose mycophenolate mofetil, an immunosuppressive drug, was associated with fewer antigen-specific B and T follicular helper (Tfh) cells after vaccination; this was not observed in patients recently infected with SARS-CoV-2. Finally, we observed that, in two independent prospective cohorts, administration of a third dose of SARS-CoV-2 mRNA vaccine restored neutralizing titers of anti-RBD IgG in about 40% of individuals who had not previously responded to two doses of vaccine. Together, these findings suggest that a third dose of SARS-CoV-2 mRNA vaccine improves the RBD-specific responses of transplant patients treated with immunosuppressive drugs.

## Teaser

Infection with SARS-CoV-2 or a three-dose mRNA vaccine regimen elicit similar neutralizing antibody responses in some kidney transplant recipients.

## INTRODUCTION

In December 2019, an outbreak of apparently viral pneumonia of unknown etiology emerged in the city of Wuhan in the Chinese province of Hubei (*1*). On 9 January 2020, the World Health Organization (WHO) announced the discovery of a novel coronavirus officially named severe acute respiratory syndrome coronavirus 2 (SARS-CoV-2), which is the pathogen responsible for coronavirus disease 2019 (COVID-19). The disease quickly disseminated from Wuhan and as at 13 January 2022, more than 307 million cases have been confirmed in 218 countries (*2*), leading the WHO to consider COVID-19 as the first pandemic triggered by a coronavirus.

Among the various alarms raised by the pandemic was its impact on the population of patients receiving organ transplants, whose COVID-19-related mortality was estimated at about 20%, several magnitudes higher than that of the general population (*3*–*7*). This vulnerable population of patients was therefore prioritized for vaccination against SARS-CoV-2 by health authorities (*8*). However, prevention of allograft rejection requires life-long immunosuppression regimens, which non-specifically inhibit T and B cells in transplant recipients, resulting in reduced response rates to vaccines in general (*9*, *10*). As expected, several recent publications have documented that immunosuppressed transplant recipients develop mitigated immune responses following the standard two-dose regimen of vaccination with either of the 2 approved SARS-CoV-2 mRNA vaccines (*11*–*15*).

Although insufficiency of vaccinal protection in transplant recipients has emerged as a concern due to accumulating reports of severe COVID-19 in vaccinated patients (*16*, *17*), the underlying immune mechanisms explaining this problem are still elusive (*15*, *18*). In an attempt to determine the relative contribution of humoral and T cell immunity in conferring protection against COVID-19 and understand immunosuppression-induced defects following SARS-CoV-2 vaccination, we undertook a prospective translational study that compared recently infected and vaccinated transplant recipients.

## RESULTS

### Infection conferred increased protection against symptomatic COVID-19 to transplant recipients relative to vaccination.

**Fig. 1.**
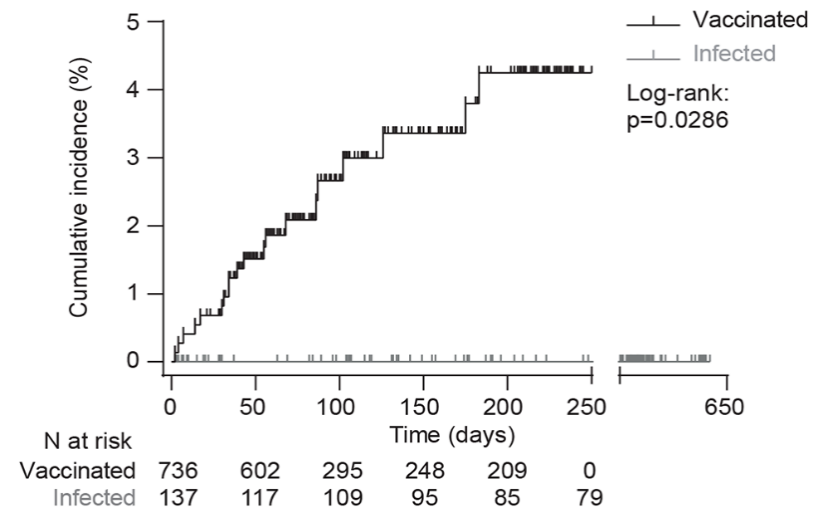
Infection confers better protection against symptomatic COVID-19 than vaccination in transplant recipients. Protection against COVID-19 was compared between renal transplant recipients with previous history of infection with SARS-CoV-2 (group “infected”, gray curve) and those who received the standard two-dose regimen of mRNA-1273 (group “vaccinated”, black curve). The follow-up started at the time of COVID-19 symptoms onset for infected patients and at the time of the second vaccine administration for the vaccinated patients. Cumulative incidence in the two groups was plotted using the Kaplan–Meier method. Data were analyzed by a Log-rank test; p=0.0286.

The incidence of COVID-19 was monitored in all 873 renal transplant recipients of Strasbourg University Hospital and compared between those with previous history of infection with SARS-CoV-2 (group “infected”, n=137) and those who received the standard two-dose regimen of vaccination with mRNA-1273 (group “vaccinated”, n=736). The clinical characteristics of this large epidemiological cohort are provided in **table S1**. Strikingly, whereas none of the recently infected patients developed symptomatic reinfection, 20 vaccinated patients developed COVID-19 (**Fig. 1**; Log-rank test, p=0.0286). Of note this observation was made during the follow-up period of recently infected patients, which was significantly longer than that of vaccinated patients (289 days, interquartile range (IQR) [119; 333] versus 79 days, IQR [56; 210], p<0.0001; Mann-Whitney test).

The total absence of symptomatic reinfection in renal transplant recipients with previous history of COVID-19 is surprising and conflicts with the results of previously published studies in the general population (*19*–*22*). However, in contrast with the previously published studies, of which two were conducted in health care workers (highly exposed to SARS-CoV-2) using systematic PCR (polymerase chain reaction) screening to define reinfection, our approach only allowed to capture symptomatic reinfections in a population particularly prone to strictly comply to social distancing rules (*23*). We concluded that SARS-CoV-2 infection confers protection against symptomatic COVID-19 to immunocompromised transplant recipients.

### Mechanistic study population details

Comparison of cellular and humoral immune responses developed by recently infected and vaccinated transplant patients offers a unique opportunity to determine which immune effector(s) are associated with protection against COVID-19 in this vulnerable population (*3*–*7*). The COVATRHUS cohort (Covid-19 Vaccine in Transplant Recipients, Hopitaux Universitaires de Strasbourg) was therefore established to prospectively collect synchronous serum and peripheral blood mononuclear cell (PBMC) samples from renal transplant recipients diagnosed with COVID-19 in absence of previous vaccine injection (group “infected”, n=21; mean sampling time: 30.6 ± 6.9 days after the onset of symptoms) or vaccinated with two doses of mRNA-1273 (group “vaccinated”, n=29; mean sampling time: 14.7 ± 3.7 days after the second dose, or 42.8 ± 3.8 days after initial contact with the antigen). This time-point for analysis was chosen based on previous studies, which reported that, in recently infected renal transplant patients, both the cellular and humoral responses against SARS-CoV-2 were clearly detectable between 25 and 37 days, although cell functionality (especially cytokine secretion) could still evolve thereafter (*24*).

The clinical characteristics of the COVATRHUS cohort are presented in **table S1**. With the exception of a shorter time post-transplantation in infected patients and a slightly different comorbidity profile of vaccinated patients, the rest of the clinical characteristics of COVATRHUS patients are similar to that of the epidemiological cohort. Recently infected and vaccinated patients from the COVATRHUS cohort had similar clinical profiles (**table S1**). Of note, the severity of COVID-19 in infected patients was mainly mild/moderate (16/21, 76%), and most of them did not require hospitalization (14/21, 67%).

### SARS-CoV-2-specific cellular immunity is comparable in previously infected and vaccinated transplant recipients.

**Fig. 2.**
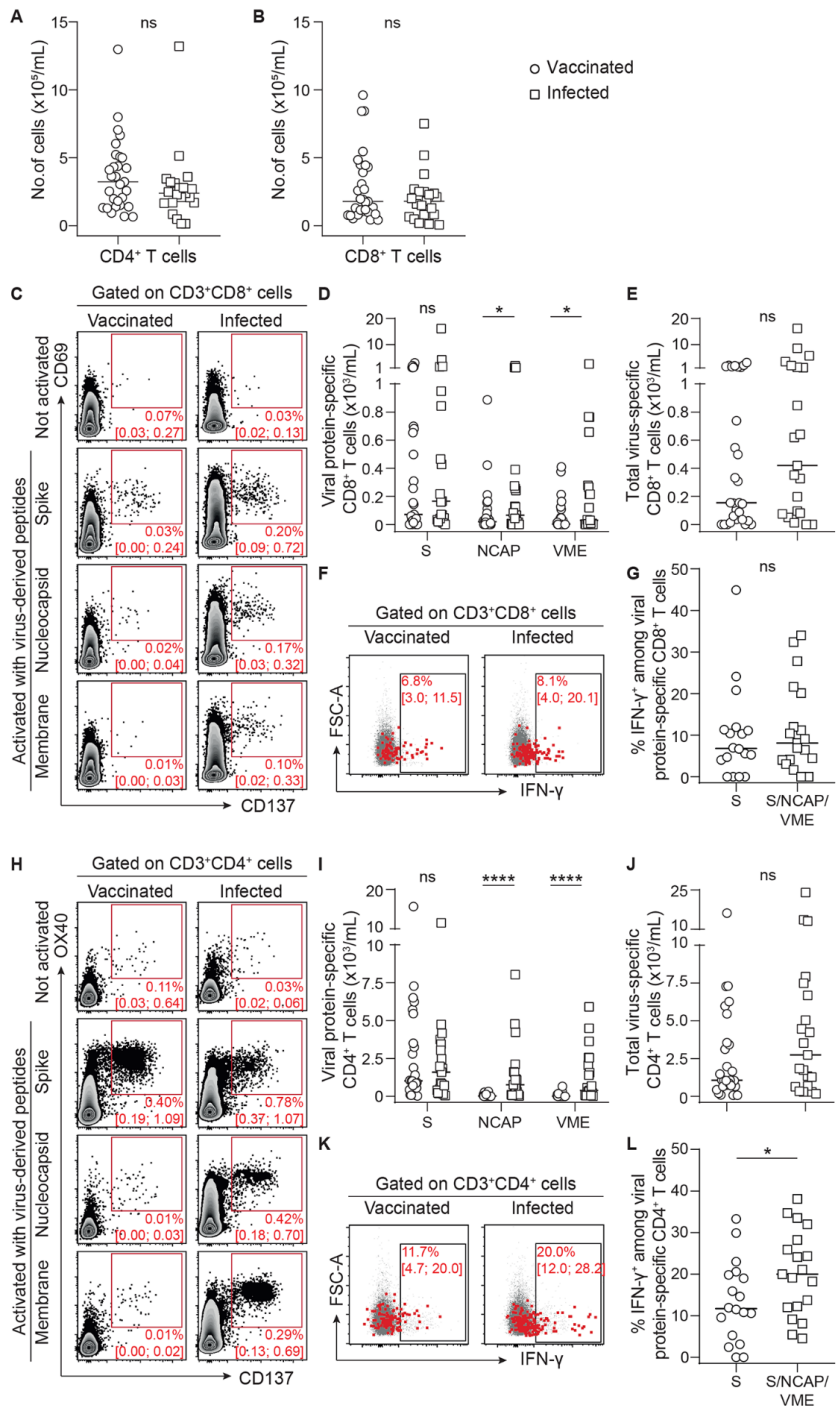
**SARS-CoV-2-specific cellular immunity was comparable in previously infected and vaccinated transplant recipients. (A** and **B)** CD4^+^ (A) and CD8^+^ (B) T cells were enumerated in the circulation of recently infected (n=21; open squares) and vaccinated (n=29; open circles) transplant recipients. (**C to L**) CD8^+^ T cells (C to G) and CD4^+^ Th1 cells (H to L) directed against the spike (S), nucleocapsid (NCAP), and membrane (VME) proteins of SARS-CoV-2 were enumerated in the circulation of recently infected and vaccinated transplant recipients. Data were background subtracted against DMSO negative control. **(C)** Flow cytometry profiles of a representative patient of each group are shown. Median percentage and interquartile range are indicated. **(D)** The count of CD8^+^ T cells specific to each viral protein is plotted for each patient. **(E)** For each patient, the total number of virus-specific CD8^+^ cytotoxic T cells is plotted. **(F)** Concatenated flow cytometry profiles of the two groups of patients are shown. Median percentage and interquartile range are indicated. FSC-A, forward scatter area. **(G)** The proportion of INFγ-producing SARS-CoV-2-specific CD8^+^ cytotoxic T cells is plotted for each patient (infected patients, n=7; vaccinated patients, n=18). **(H)** Flow cytometry profiles of a representative patient of each group are shown. Median percentage and interquartile range are indicated. **(I)** The count of Th1-polarized CD4^+^ T cells specific to each viral protein is plotted for each patient. **(J)** For each patient, the total number of virus-specific Th1-polarized CD4^+^ T cells is plotted. **(K)** Concatenated flow cytometry profiles of the two groups of patients are shown. Median percentage and interquartile range are indicated. **(L)** The proportion of IFN-γ-producing SARS-CoV-2-specific Th1 CD4^+^ T cells is plotted for each patient (infected patients, n=7; vaccinated patients, n=18). The bars indicate the median. Data were analyzed using a Mann-Whitney test; ns, p>0.05; *p≤0.05; ****p<0.0001.

Virus-specific CD8^+^ T cells reduce disease severity and promote recovery in many respiratory infections, including those driven by coronaviruses (*25*, *26*), by eliminating infected cells. Optimal generation of these cytotoxic effectors depends upon the help provided by the Th1 CD4^+^ T cells (*27*). We observed no difference in the total count of CD4^+^ and CD8^+^ T cells was observed between vaccinated and recently infected patients (**Fig. 2, A and B**). Cytotoxic CD8^+^ T cells directed against the spike protein of SARS-CoV-2, identified by the co-expression of CD69 and CD137 (*28*), could be detected in the circulation of both vaccinated and recently infected patients (**Fig. 2C**). However, only recently infected patients had CD8^+^ T cells directed against the other proteins of the virus (nucleocapsid and membrane). This finding was expected since nucleocapsid and membrane proteins are not included in the vaccine formulation (**Fig. 2, C and D****).** There was no difference in spike protein-specific CD8^+^ T cells in the circulation of patients with recent infection versus vaccinated patients (**Fig. 2D**). The result remained the same when all specificities (spike, nucleocapsid, and membrane) were added together to better take into account the difference of repertoire between the two groups (**Fig. 2E**). Importantly, the functionality of these SARS-CoV-2-specific CD8^+^ T cells was demonstrated by their ability to produce interferon gamma (IFN-γ) upon in vitro stimulation (**Fig. 2F****)**. The frequency of IFN-γ−producing SARS-CoV-2-specific CD8^+^ T cells was similar between vaccinated and recently infected patients (**Fig. 2G****)**.

SARS-CoV-2-specific CD4^+^ T cell responses were monitored using the same approach as above (*28*); OX40 and CD137 were used as surface activation-induced markers on CD4^+^ T cells (**Fig. 2H**). Comparison of CD4^+^ and Th1 responses of vaccinated and recently infected patients resulted in the same conclusions as for CD8^+^ T cell responses (**Fig. 2****, I to L**).

Thus, although the repertoire of the cellular immune response directed against SARS-CoV-2 is wider in recently infected patients (**Fig. 2C and D****, ****2H to I**), the minimal increase in cellular effectors (p=0.240 for CD8^+^ T cells, **Fig. 2E** and p=0.158 for CD4^+^ T cells, **Fig. 2J**) is unlikely to account alone for the drastic advantage in term of protection against symptomatic COVID-19 observed in this group as compared with vaccinated transplant recipients. Another argument in favor of this hypothesis is the fact that some recently infected patients had barely detectable virus-specific T cells, suggesting that their protection was due to other types of immune effectors, a hypothesis also supported by a recently published experimental study (*29*).

### Presence of neutralizing IgG correlates with protection against COVID-19 in transplant recipients

**Fig. 3.**
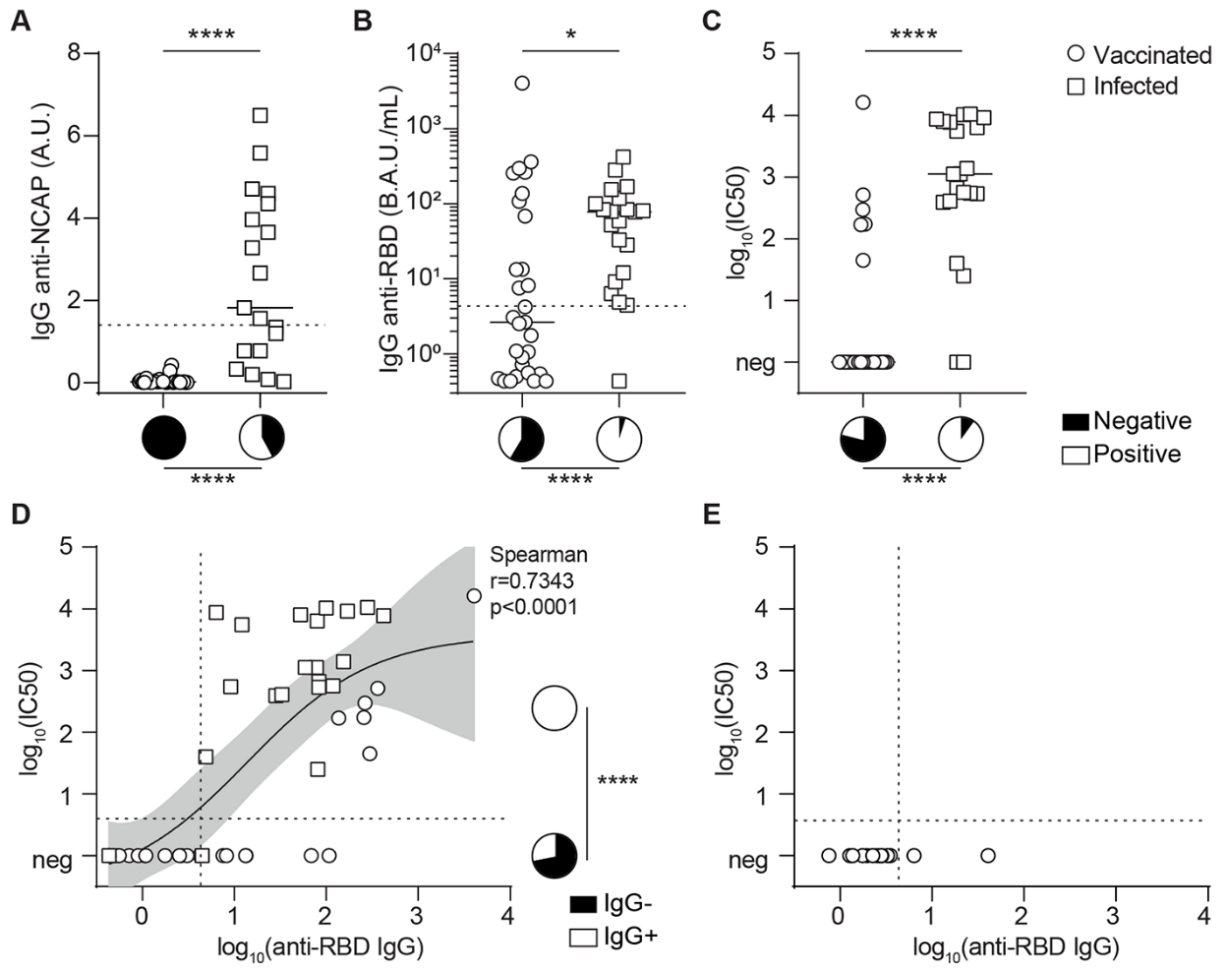
Anti-SARS-CoV-2 specific humoral immunity elicited by infection and vaccination differ in transplant recipients. **(A and B)** The titers of IgG antibodies directed against the nucleocapsid protein (A) or receptor binding domain (B) of SARS-CoV-2 were measured in the circulation of recently infected (n=21; open squares) and vaccinated (n=29; open circles) transplant recipients. A.U., arbitrary units; B.A.U., binding antibody units. **(C).** The neutralizing capacity of patients’ serum was compared between recently infected (n=21; open squares) and vaccinated (n=29; open circles) transplant recipients. Neutralizing titers are presented as the log 10 of the dilution inhibiting 50% of target infection, or log_10_(IC50). Neg indicates no evidence of neutralizing antibodies. For (A to C), the bars indicate median values. Pie charts are used to compare proportions. **(D and E)** The values of anti-RBD IgG titers and neutralizing capacity of the serum were log-transformed and plotted. **(D)** Results for the patients of the COVATRHUS cohort who were infected (n=21; open squares) or vaccinated (n=29; open circles) are plotted. The relation between the two variables was analyzed with a non-linear regression model using a 4 parameters slope. The result of Spearman correlation test is shown on the graph. The pie charts represent the proportion of patients with (white) anti-RBD IgG among those with or without neutralizing humoral response. **(E)** Results for the 14 patients from the epidemiological cohort, who developed COVID-19 after vaccination, are plotted. Dotted lines indicate the threshold of positivity of each assay. Mann-Whitney tests were used to compare antibody or neutralizing titers in (A to C) and Fisher’s exact test was used to compare proportions in (A to D); *p≤0.05; ****p<0.0001.

Beside cellular effectors, the adaptive immune system also generates antibodies against SARS-CoV-2. As expected, antibodies directed against viral nucleocapsid (not included in the vaccine formulation) were exclusively detected in patients from the recent infection group (**Fig. 3A**), but only in half of them (11/19, 58%). In contrast almost all (20/21, 95%) recently infected transplant recipients developed anti-RBD (Receptor-Binding Domain) IgG (**Fig. 3B**). The spike glycoprotein mediates virus entry into target cells using the Angiotensin-Converting Enzyme 2 (ACE2) receptor, and it has been shown that antibodies directed against the RBD can block viral infection of human cells in vitro and counter viral replication in vivo (*30*–*34*). Despite the fact that anti-RBD IgG titers were lower than those observed in a cohort of 30 vaccinated healthy volunteers (*35*), serum isolated from recently infected transplant recipients still efficiently block pseudo-virus entry in human cells in vitro (**Fig. 3C**). A positive correlation between anti-RBD IgG titer and the result of the in vitro neutralization assay was demonstrated (**Fig. 3D**). In contrast with recently infected patients, the humoral response of the vaccinated group against RBD was heterogeneous, and most patients (17/29, 59%) failed to generate detectable anti-RBD IgG after two doses of vaccine (**Fig. 3B**). This defect was even more clear in the context of a pseudo-virus neutralization assay, in which only 21% of vaccinated patients had neutralizing antibody response against the pseudo-virus (6/29; **Fig. 3C**).

These findings led us to hypothesize that the lack of protection against COVID-19 in some vaccinated transplant recipients may be due to insufficient generation of neutralizing anti-RBD antibodies. To test this theory, we retrieved the 14 available serum samples collected after the two doses of mRNA-1273 but prior COVID-19 diagnosis for the vaccinated patients of the epidemiological cohort. In line with our hypothesis, only 2/14 (14%) patients had detectable circulating anti-RBD IgG antibodies after the standard scheme of vaccination and none of these serum samples were able to block the entry of pseudo-virus in human cells in vitro (**Fig. 3E**). Thus, the 29 vaccinated transplant recipients were distributed into the group “responder” (n=6/29, 21%) or “non-responder” (n=23/29, 79%) to vaccine according to whether or not serum collected after two doses of mRNA-1273 vaccine showed neutralizing capacity against pseudo-virus in vitro. Clinical and biological characteristics of these two groups are similar and presented in **Table 1**.

**Table 1.** Characteristics of vaccinated patients.

**n (%) or median [IQR]**	**Non-responders** **N = 23**	**Responders** **N = 6**		p*
**Age (y)**	61.2 [45.8; 70.1]	47.7 [41.2; 61.7]		0.254
**Male**	14 (61)	4 (67)		>0.999
**BMI**	24.9 [23.8; 29.4]		23.8 [20.2; 24.5]	0.138
**Comorbidities** Cardiopathy Diabetes	15 (65) 3(13)		6 (100) 0 (0)	0.138 >0.999
**Time since transplantation (y)**	7.0 [1.6; 15.9]		10.4 [3.5; 24.6]	0.414
**Donor type** Deceased Living	20 (87) 3 (13)	5 (83) 1 (17)		>0.999
**Induction therapy** Anti-thymocyte globulins Basiliximab No induction NA	13 (57) 8 (35) 1 (4) 1 (4)	4 (67) 1 (17) 1 (17) 0 (0)		0.453
**Maintenance immunosuppression** CNI (yes) MMF/MPA (mg/day) Steroids (mg/day) imTOR (yes) Belatacept (yes)	22 (96) 1000 [500; 1000] 5.0 [0.0; 5.0] 1 (4) 1 (4)	6 (100) 250 [0; 625] 2.5 [0.0; 5.0] 2 (33) 0 (0)		>0.999 **0.014** 0.358 0.100 >0.999
**Biological data** Lymphocytes (G/L) Monocytes (G/L) CRP (mg/L) Albumin (g/L) Creatinine (μmol/L)	1.16 [0.99; 1.38] 0.55 [0.41; 0.79] 4.0 [4.0; 5.8] 43 [42; 46] 134 [97; 183]	1.99 [1.45; 2.66] 0.51 [0.44; 0.70] 4.5 [4; 10.6] 44 [43; 45] 131 [97; 237]		0.069 0.723 0.570 0.874 0.859

*qualitative variables were compared using a Fisher or Chi-square test, quantitative variables were compared using a Mann-Whitney test. IQR: interquartile range; y, years; BMI, body mass index; NA, not available; CNI: calcineurin inhibitor; MMF/MPA: mycophenolate mofetil/mycophenolic acid; imTOR, inhibitor of the mechanistic target of rapamycin; CRP, C-reactive protein.

### Generation of neutralizing antibodies following vaccination was associated with evidence of germinal center derived B cell responses.

The immunologic dogma has long held that the generation of IgG against protein antigen was dependent upon complex interactions between antigen-specific B cells and cognate CD4^+^ T follicular helper (Tfh) cells that take place in specialized structures of secondary lymphoid organs called germinal centers (*36*, *37*). However, this has been challenged by a number of studies (*38*–*41*). It is now clear that IgG can be generated during extrafollicular responses (which are sometimes independent of T cells).

**Fig. 4.**
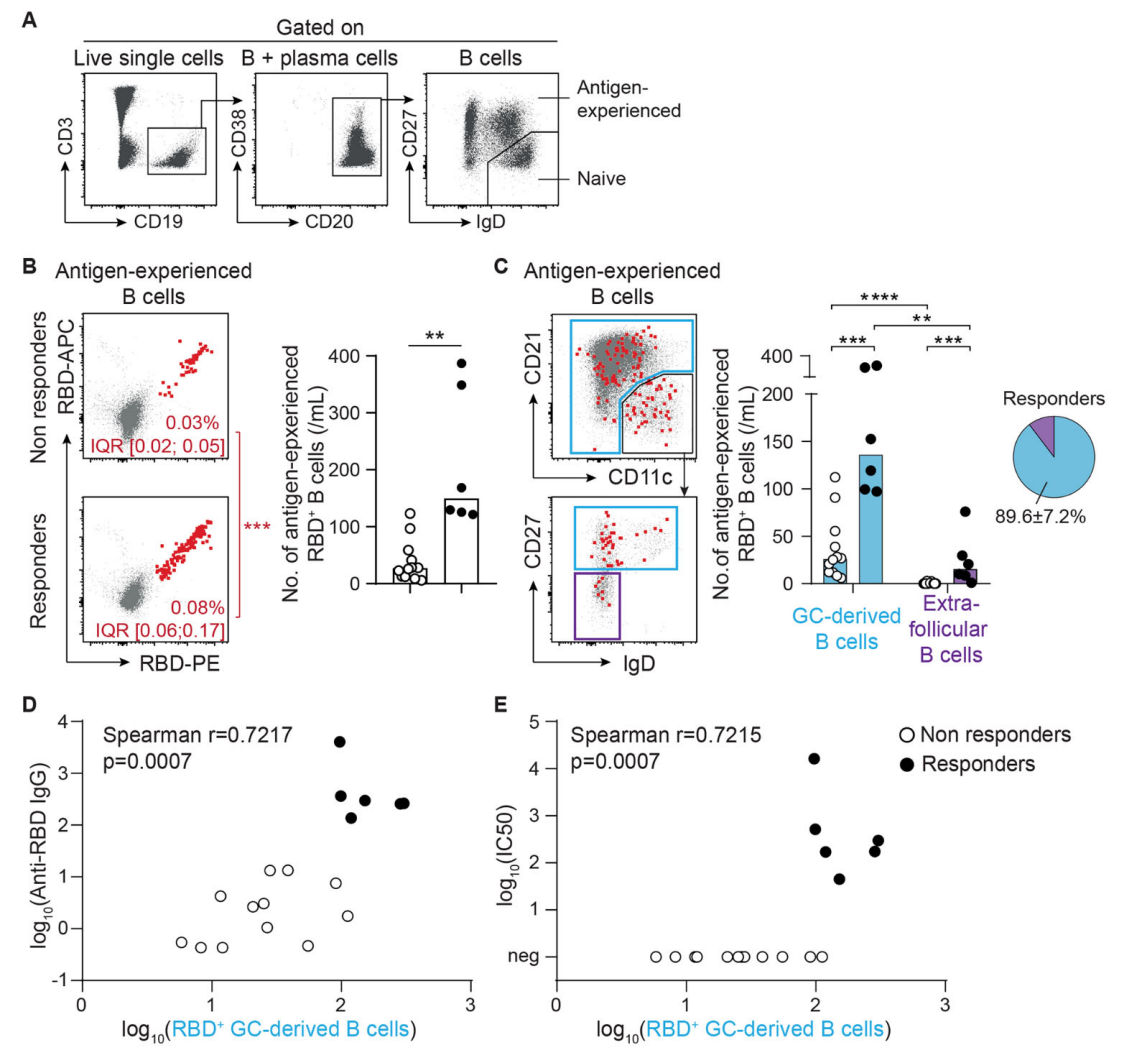
**Generation of neutralizing IgG antibodies after vaccination is associated with evidence of a germinal center reaction. (A)** The gating strategy used for flow cytometry analysis of RBD-specific B cell response is shown. **(B)** RBD-specific cells were enumerated among antigen-experienced B cells in the circulation of vaccinated renal transplant recipients. Left panel: concatenated flow cytometry profiles of non-responders (upper thumbnail) and responders (lower thumbnail) to vaccine are shown. Median percentage and interquartile range are indicated. Proportions of RBD-specific B cells were compared. Right panel: the numbers of RBD-specific antigen-experienced B cells of non-responders (n=12; open circles) and responders (n=6; black circles) were compared. **(C)** The site in which the humoral response against the vaccine developed was indirectly analyzed based upon the phenotype of RBD-specific B cells. Extrafollicular responses are characterized by the generation of type 2 double-negative (CD11c^high^ CD21^low^ IgD^-^ CD27^-^) B cells (purple gate). The rest of antigen-experienced B cells (blue gates) are thought to be derived from the germinal center. Left panels: concatenated flow cytometry profiles of all vaccinated patients are shown, together with the gating strategy used for analysis. Right panel: Bar graphs (left) show the number of RBD-specific antigen-experienced B cells likely derived from germinal centers (GC, blue) or extrafollicular (purple) responses for non-responders (n=12; open circles) and responders (n=6; black circles) to vaccine. The proportions of RBD-specific antigen-experienced B cells derived from germinal center and extrafollicular responses in responders to vaccine are shown in the pie chart (right). Data in (B and C) were analyzed using a Mann-Whitney test. **p<0.01; ***p<0.001; ****p<0.0001. **(D and E)** The correlation between the number of germinal center-derived RBD-specific antigen-experienced B cells and the titer of anti-RBD IgG (**D**) or the viral neutralization capacity of the serum (**E**) are shown. The results of Spearman correlation test are shown on the graphs.

To characterize where IgG response to COVID-19 mRNA vaccine develops, RBD-specific B cells were enumerated in the circulation of vaccinated patients and their expression of CD21, CD11c, CD27 and IgD was determined by flow cytometry (**Fig. 4, A to C**). Previous studies have demonstrated that the extrafollicular differentiation pathway generates an atypical population of antigen-experienced B cells that is referred to as type 2 double-negative (IgD^-^CD27^-^). The latter is characterized by high expression of CD11c and low to negative expression of CD21 (*42*, *43*). As such, they differ from their conventional germinal center-derived counterparts which are mostly CD27^+^. As expected, RBD-specific B cells were found in both higher proportion and number in the circulation of responders than non-responders to vaccine (**Fig. 4B**). B cells expressing a germinal center-associated phenotype represented the vast majority (about 90%) of RBD-specific B cells in the circulation of responders to vaccine (**Fig. 4C**). Furthermore, their number correlated well with both the anti-RBD IgG titers (**Fig. 4D**) and the in vitro viral neutralization capacity of their serum (**Fig. 4E**).

### Generation of neutralizing antibodies after vaccination is associated with circulating spike protein-specific Tfh cells.

**Fig. 5.**
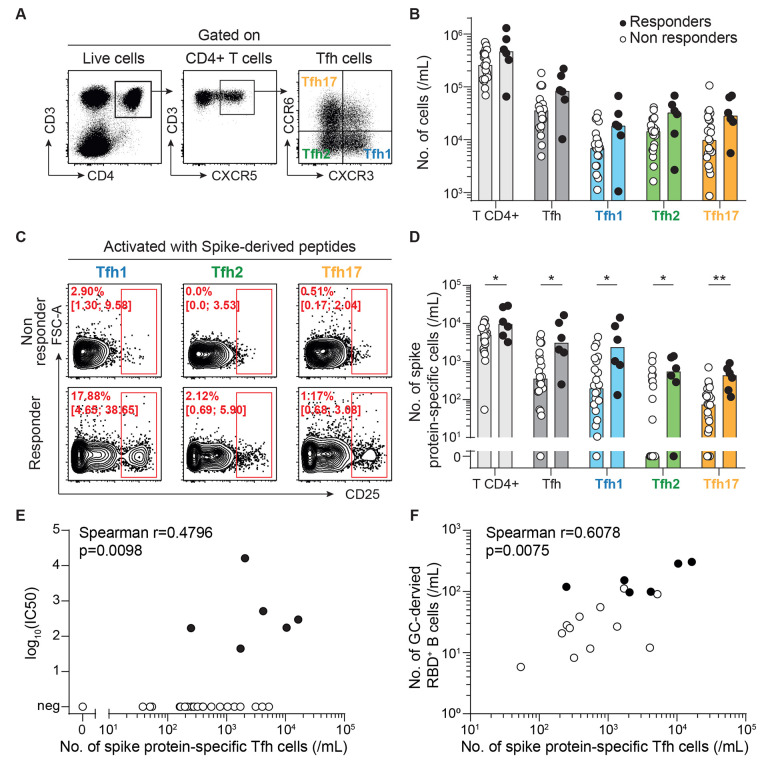
Generation of neutralizing antibodies after vaccination correlates with the number of spike protein-specific Tfh cells. Follicular helper T cells (Tfh) were enumerated in the circulation of responders (n=6; black circles) and non-responders (n=22; open circles) after two doses of SARS-CoV-2 mRNA vaccine. **(A)** Representative flow cytometry profiles are shown with the gating strategy used to identify the 3 subsets of follicular helper T cells (Tfh): Tfh1 (blue), Tfh2 (green), and Tfh17 (orange). **(B)** The counts of circulating CD4^+^ T cell subsets are plotted for each patient. **(C and D)** Spike protein-specific cells were enumerated among each CD4^+^ T cell subset for each vaccinated patient. Data were background subtracted against a DMSO-only negative control. **(C)** Representative flow cytometry profiles of non-responders (upper row) and responders (lower row) are shown. Median percentage and interquartile range are indicated. **(D)** The counts of circulating spike protein-specific CD4^+^ T cell subsets are plotted for each patient. Bars indicate median values. Data in (D) were compared using Mann-Whitney tests. *p<0.05; **p<0.01. **(E)** The correlation between the number of spike protein-specific Tfh cells and viral neutralization capacity of the serum is shown. The result of Spearman correlation test is shown on the graph. **(F)** The correlation between the number of spike protein-specific Tfh cells and germinal center-derived RBD-specific antigen-experienced B cells is shown. The result of Spearman correlation test is shown on the graph.

Since the humoral response to SARS-CoV-2 correlated with abundance of B cells expressing a germinal center-associated phenotype, we speculated that a Tfh defect may be contributing to the lack of generation of neutralizing antibodies observed in some vaccinated transplant recipients, an hypothesis justified by the detrimental impact of maintenance immunosuppression on Tfh functions (*9*, *36*, *44*). Although Tfh cells act within germinal centers in secondary lymphoid organs, recent studies have demonstrated that human blood CXCR5^+^CD4^+^ T cells are counterparts of Tfh. This population contains specific subsets that differentially support antibody secretion and can be identified on the basis of their profile of chemokine receptor expression (*45*). In line with these studies, the 3 subsets of Tfh, Tfh1 (CXCR3^+^CCR6^-^), Tfh2 (CXCR3^-^CCR6^-^), and Tfh17 (CXCR3^-^CCR6^+^), could be identified and enumerated by flow cytometry in the circulation of vaccinated patients (**Fig. 5A**). No difference was observed regarding the total count of CD4^+^ T cells, Tfh, or any of the Tfh subsets between responders and non-responders (**Fig. 5B**). However, in line with our hypothesis, all subsets of spike protein-specific CD4^+^ T cells were found in higher quantity in the circulation of responders than non-responders (**Fig. 5, C and D**). Moreover, a positive correlation between the total number of spike protein-specific Tfh and the neutralizing capacity of the sera was observed (**Fig. 5E**). This observation remained true when sub-analyses were conducted separately with the 3 different subsets of Tfh (**fig. S1**).

Finally, a strong positive correlation was also observed between the number of germinal center-derived RBD-specific B cells and that of cognate Tfh cells (**Fig. 5F**), further emphasizing the importance of bidirectional interactions between these partners within the germinal center for an efficient response to SARS-CoV-2 vaccine.

### High mycophenolate mofetil dose was associated with reduced vaccine response in vaccine recipients.

**Fig. 6.**
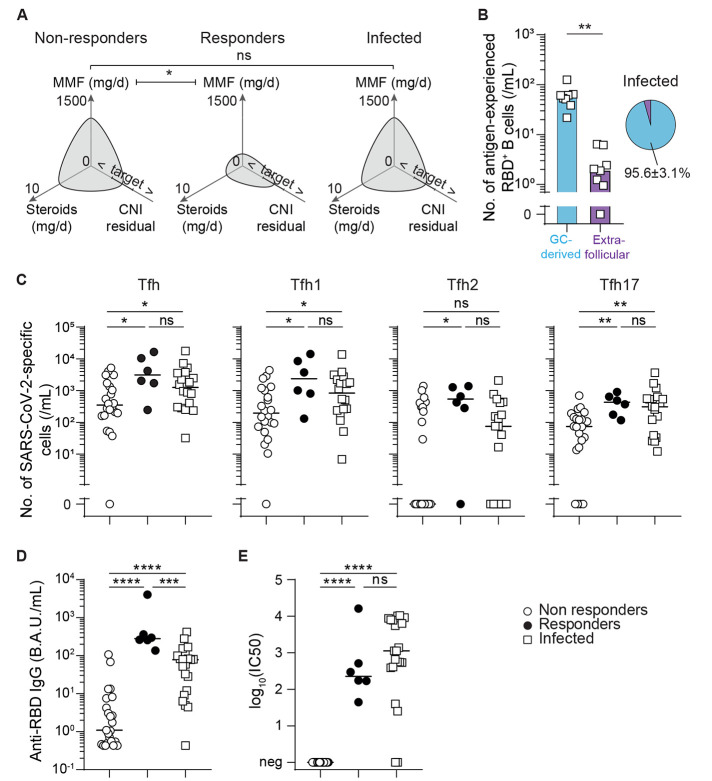
**High mycophenolate mofetil dose was associated with evidence of poorer vaccination-induced germinal center reactions. (A)** Polar plots were used to compare maintenance immunosuppression regimens for non-responders (n=23; left panel) and responders (n=6; middle panel) to two doses of SARS-CoV-2 mRNA vaccine, and patients previously infected with SARS-CoV-2 (n=21; right panel). Median values are plotted. MMF, mycophenolate mofetil; CNI, calcineurin inhibitor. Target indicates the target residual blood concentration of CNI. < and > symbols indicate residual blood concentrations of CNI below or above the target, respectively. **(B)** The bar graph (left) shows the number of RBD-specific antigen-experienced B cells thought to be derived from germinal center (blue) and extrafollicular (purple) responses of each recently infected patient (n=8; open squares). The proportions of RBD-specific antigen-experienced B cells likely derived from germinal center and extrafollicular responses in recently infected patients are shown in the pie chart (right). **(C)** SARS-CoV-2-specific Tfh subsets were enumerated in the circulation of non-responders (n=22; open circles) and responders (n=6; black circles) to two doses of SARS-CoV-2 mRNA vaccine, as well as for patients recently infected with SARS-CoV-2 (n=18; open squares). **(D)** The titers of anti-RBD antibodies were measured in the circulation for non-responders (n=23; open circles) and responders (n=6; black circles) to two doses of SARS-CoV-2 mRNA vaccine, as well as for patients recently infected with SARS-CoV-2 (n=21; open squares). B.A.U. indicates binding antibody units. **(E)** The neutralizing capacity of patients’ serum was compared for non-responders (n=23; open circles) and responders (n=6; black circles) to two doses of SARS-CoV-2 mRNA vaccine, as well as for patients recently infected with SARS-CoV-2 (n=21; open squares). Neutralizing titers are presented as log_10_(IC50). Bars indicates the median. Data were analyzed by Mann-Whitney tests; ns, p>0.05; *p≤0.05, **p<0.01; ***p<0.001, ****p<0.0001.

The dynamic of germinal center reactions, in which antigen-specific B and T cells proliferate, is the major determinant controlling the humoral immune response after vaccination against SARS-CoV-2 in healthy volunteers (*46*, *47*). The reduced count in both spike protein-specific B and Tfh cells observed in non-responders to vaccine therefore provides a potential explanation for the defect of generation of anti-RBD IgG and in turn, the lack of viral neutralization capacity of their serum. We next asked what distinguished non-responders from responders in our vaccinated cohort. Among the immunosuppressive drugs used in maintenance regimen, some block the activation of T cells (calcineurin-inhibitor) whereas others, such as mycophenolate mofetil, act by blocking the proliferation of adaptive immune effectors. Although responders and non-responders to vaccine were similarly exposed to calcineurin-inhibitors, non-responders received significantly more mycophenolate mofetil (250mg/day, IQR [0,625] versus 1000mg/day, IQR [500; 1000] in responders versus non-responders, p=0.014; **Fig. 6A**).

This result suggests that the anti-proliferative effect of high dose mycophenolate mofetil may impede germinal center reaction and thereby be the cause of the lack of response after two doses of mRNA-1273 vaccine observed in some transplant recipients. However, despite the fact that recently infected patients received the same (high) dose of mycophenolate mofetil at the time of infection as non-responders to vaccine (**Fig. 6A**), they generated higher numbers of virus-specific germinal center-derived B cells (**Fig. 6B**) and Tfh (**Fig. 6C**), and consequently neutralizing anti-RBD IgG antibodies, as do responders to vaccine (**Fig. 6, D and E**).

### A third dose of mRNA vaccine improved neutralizing anti-RBD IgG responses in a subset of prior vaccine non-responders.

**Fig. 7.**
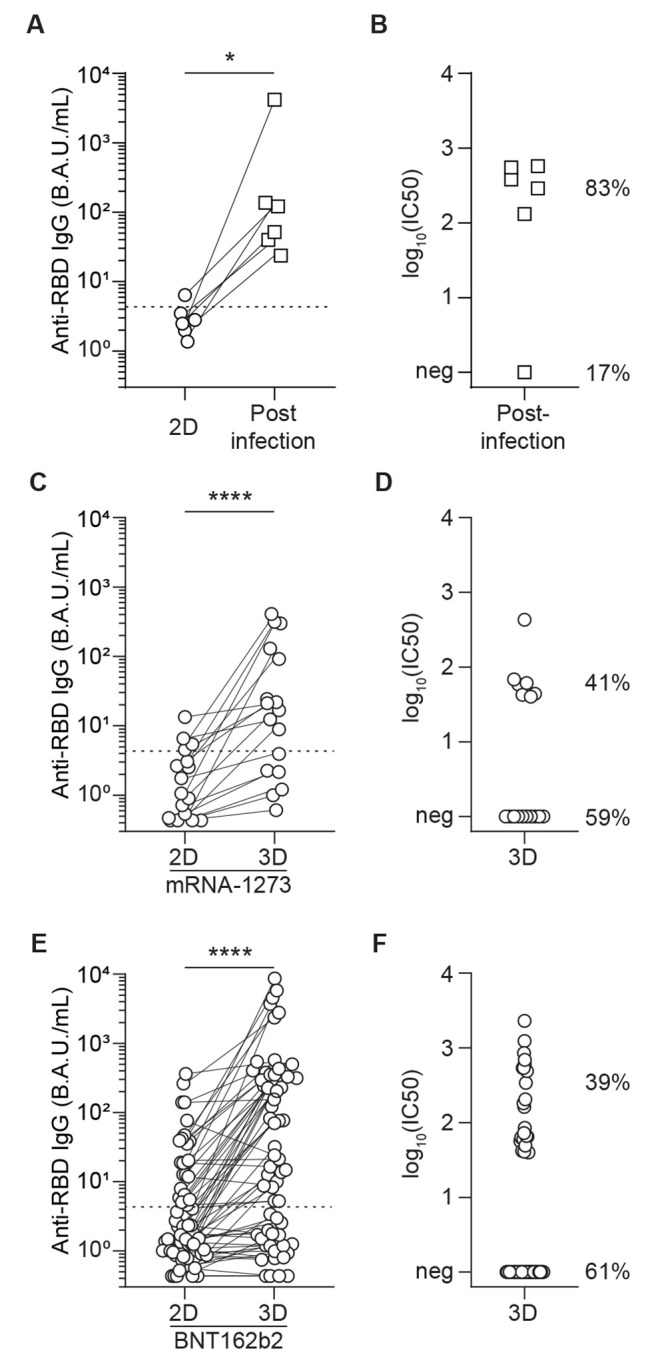
**Infection after vaccination or a third dose of mRNA vaccination improves SARS-CoV-2-specific antibody responses. (A)** Anti-RBD IgG titers were measured pre- and post-SARS-CoV-2 infection in individuals who received two doses (2D) of mRNA vaccine (n=6). **(B)** Virus neutralization capacity of the serum was measured after SARS-CoV-2 infection in transplant recipients who did not respond to two doses of mRNA vaccine (n=6). Percentages indicate the fraction of individuals with (83%) or without (17%) measurable neutralizing titers after two doses of vaccine and SARS-CoV-2 infection. **(C)** A discovery cohort (mRNA-1273 vaccine; n=17) was used to compare anti-RBD IgG titers after the second (2D) and third (3D) dose of mRNA vaccine in the same patients; these patients were considered non-responders after two doses of mRNA-1273 vaccine. **(D)** Virus neutralization capacity of the patients’ serum was measured after 3D (n=17). Percentages indicate the fraction of individuals with (41%) or without (59%) measurable neutralizing titers after three doses of mRNA-1273 vaccine. **(E)** An external validation cohort (BNT162b2 vaccine; n=62) was used to compare anti-RBD IgG titers after the second (2D) and third (3D) dose of mRNA vaccine in those who were non-responders to two doses of BNT162b2 mRNA vaccine. **(F)** Virus neutralization capacity of the patients’ serum was measured after 3D (n=62). Percentages indicate the fraction of individuals with (39%) or without (61%) measurable neutralizing titers after three doses of BNT162b2 vaccine. Wilcoxon test; *, p<0.05, ****, p<0.0001.

Our last observation led us to ask if the potential negative impact of high dose mycophenolate mofetil could be overcome by further immunogenic stimulation than the standard vaccination scheme, such as the one provided to the patients by infection with live virus. In line with this hypothesis, vaccinated patients without neutralizing anti-RBD IgG after two doses of mRNA-1273 did generate neutralizing anti-RBD IgG after infection (**Fig. 7, A and B**). Based on these results, we tested the impact of an additional dose of vaccine on the humoral response of 17 of the 23 transplant patients that were non-responders to the standard two-dose vaccine regimen for mRNA-1273. In accordance with our hypothesis, we not only observed an increase in anti-RBD IgG titers after the third dose of vaccine (**Fig. 7C**), but more importantly, 41% of the serum samples (7/17) efficiently block pseudo-virus entry in human cells in vitro (**Fig. 7D**).

We next validated these findings in an independent, external cohort. A third dose of the other currently-approved mRNA SARS-CoV-2 vaccine (BNT162b2) was administered to a cohort of 62 renal transplant recipients from Lyon University Hospital that did not have neutralizing anti-RBD IgG after 2 doses of vaccine. In accordance with our previous results with mRNA-1273, we observed a similar increase in anti-RBD IgG titers in these non-responders after the third dose of vaccine (**Fig. 7E**), and serum from 39% (24/62) of recipients efficiently blocked pseudo-virus entry in human cells in vitro (**Fig. 7F**).

## DISCUSSION

Although antibody titers and their ability to neutralize the virus are emerging as correlates of protection against COVID-19 in healthy individuals (*48*–*50*) there is still an urgent need to understand the relative contribution of humoral and T cell immunity in conferring protection to immunosuppressed populations (*18*), in particular transplant patients, who are both at high risk of death due to COVID-19 (*3*–*7*) and poor responders to mRNA vaccines (*11*–*14*, *51*).

Taking advantage of the observation that a previous infection by SARS-CoV-2, but not the standard two-dose scheme of vaccination, provided protection against symptomatic COVID-19 to transplant recipients, we designed a translational study to compare the adaptive immune responses of these two groups of patients. The results of this study suggest that germinal center-derived anti-SARS-CoV-2 neutralizing IgG may be a critical component of the adaptive immune response associated with protection against symptomatic COVID-19 in transplant recipients. Our data also suggest that the negative impact of mycophenolate mofetil on response to vaccine may be overcome by increasing antigen exposure with a third dose.

Newer studies have challenged a long-standing dogma in immunology, which considered switched antibodies directed against protein antigens (such as spike protein) a hallmark of germinal center reactions. For example, recent experimental works have demonstrated that T cell-independent IgG class switching can also occur, in particular against certain outer membrane proteins of pathogens (*39*, *40*). Additional studies have shown that, during T cell-dependent humoral response, IgG class switching is triggered prior to differentiation into germinal center B cells (*38*). Finally, it has been demonstrated that IgG class-switching can also take place during an extrafollicular (and thus germinal center-independent) differentiation pathway that is promoted by inflammatory conditions (*41*), including in the particular setting of severe COVID-19 (*52*). Our observation that the generation of virus-neutralizing IgG in responders to vaccination correlated with both the number of antigen-specific germinal center B cells and Tfh cells may indicate that the response to SARS-CoV-2 mRNA vaccine require germinal center reactions in renal transplant recipients, as has been recently reported for immunocompetent healthy volunteers (*46*).

Interestingly, serum neutralization capacity and antigen-specific germinal center B cells after vaccination do not only correlate with the number of antigen-specific Tfh1 cells, the subset predominantly produced after vaccination in healthy participants (*53*, *54*), but also with the two other Tfh subsets (Tfh2 and Tfh17). These populations are thought to be the most efficient to drive antibody generation (*9*, *45*). This latter finding, which conflicts with the negative correlation recently reported after infection between the number of Tfh17 cells and the neutralizing antibody response (*55*), could indicate that efficient germinal center response to infection and vaccination require different Tfh subpopulations.

It is not clear what factors impair germinal center reaction in non-responders to SARS-CoV-2 mRNA vaccine. We observed that transplant patients without viral neutralizing IgG after two doses of vaccine were exposed to higher dose of mycophenolate mofetil, an immunosuppressive drug that acts by blocking proliferation of activated B and T lymphocytes (*56*, *57*). This observation is supported by other independent studies, which have also reported an association between exposition to mycophenolate mofetil and lower antibody responses (*44*, *58*, *59*), including to SARS-CoV-2 vaccines (*11*, *14*). Based on these findings, it is tempting to speculate that a reduction (or suspension) of the maintenance dose of mycophenolate mofetil prior vaccination might help obtaining better response rates. On the other hand, this non-antigen specific attitude might increase the risk of generation of donor-specific antibodies (*9*), which is the first cause of late allograft loss (*60*) through accelerated chronic vascular rejection (*61*, *62*).

Based on the observation that recently infected patients successfully generated viral neutralizing IgG despite high dose of mycophenolate mofetil, similar to that of non-responders, we hypothesized that an additional exposure to viral antigen in the form of a third dose of vaccine could improve a patient’s protection without requiring the reduction of maintenance immunosuppression. In line with this hypothesis, administration of a third dose of mRNA vaccine indeed resulted in the generation of neutralizing anti-RBD IgG in about 40% of individuals who did not respond to the standard two-dose course of vaccination. This result was further validated in a larger independent prospective cohort with the other approved SARS-CoV-2 mRNA vaccines and has been reported by independent groups (*15*, *63*–*66*). Furthermore, our group recently reported that a fourth dose of an mRNA-based vaccine produces a satisfactory antibody response in some kidney transplant recipients who did not respond adequately after 3 previous doses (*67*).

In addition to increasing the number of vaccinations, another possibility to increase vaccine immunogenicity is to increase the amount of antigen provided in each dose. This strategy has been successfully tested in transplant recipients with protein-based vaccines against influenza (*68*, *69*). In this regard, it is interesting to note that several studies have already reported higher antibody titers in healthy patients vaccinated with mRNA-1273 (which contains 100μg of mRNA) than in those that received BNT162b2 (30μg of mRNA) (*70*), though whether one vaccine is more effective than the other in this cohort remains to be evaluated.

The process of adapting vaccination regimens has limits. A fraction of transplanted patients will likely not be able to generate an efficient antibody response whatever the vaccination scheme. In this cohort, protection against COVID-19 might depend on infusion of cocktails of therapeutic or prophylactic mAbs. This primary prevention strategy has indeed been successfully tested in people with household exposure to SARS-CoV-2 with the combination of casirivimab and imdevimab (REGEN-COV) (*71*). In this study, mAbs infusion reduced the risk of developing symptomatic and asymptomatic COVID-19 and also reduced the duration of symptoms. Further studies evaluating this strategy of passive immunization in organ transplant recipients are essential to protecting this at-risk population.

This study has several limitations. First, only a limited number of patients were enrolled (n=50), the immune response of whom was analyzed at only a single timepoint. Second, the impact of vaccination or infection on the various immune cell subsets was analyzed in peripheral blood instead of the secondary lymphoid organs (spleen and lymph nodes), where immune responses actually develop. This limitation made it impossible to directly evaluate the formation of germinal centers in responders to vaccines and during mild-to-moderate COVID-19 disease. Third, these data were collected prior to the emergence of the omicron variant, which is currently the dominant circulating variant of concern. Finally, we did not directly test the hypothesis that stopping mycophenolate mofetil would allow for better expansion of antigen-specific B and T cells and thereby an improved response rate to vaccination. Thus, future studies to investigate causal relationships between these parameters are needed.

In conclusion, our study suggests that the protection of renal transplant recipients against severe COVID-19 depends upon the germinal center-dependent generation of virus-neutralizing IgG antibodies. In contrast with SARS-CoV-2 infection, which efficiently drives protective humoral response, the standard two-dose regimen of mRNA vaccine might be insufficient in some transplant patients treated with immunosuppressive drugs. Thus, these patients may require additional booster dose(s) of mRNA vaccine.

## MATERIALS AND METHODS

### Study design

A monocentric epidemiological cohort of kidney transplant patients was used to retrospectively compare the incidence of symptomatic SARS-CoV-2 infections in patients vaccinated against SARS-CoV-2 with two doses of mRNA vaccine versus patients with a previous history of COVID-19. A cohort of 50 patients (21 recently infected and 29 vaccinated, COVATRHUS cohort) was extracted from this initial cohort for in depth retrospective analysis of their cellular and humoral immune responses against SARS-CoV-2. The impact of a third dose of mRNA vaccine was first evaluated in the non-responders of COVATRHUS cohort (n=23) and then in an external validation cohort (n=62) in a prospective observational study.

### Characteristics of study populations

The incidence of SARS-CoV-2 infections was monitored since the beginning of the pandemic, in the entire cohort of kidney transplant recipients at the University Hospital of Strasbourg, France, and compared between patients with a previous history of COVID-19 and those who received the two doses of mRNA-1273. The follow-up started at the time of COVID-19 symptoms onset for the infected patients. For vaccinated transplant recipients, since the protection conferred by mRNA vaccine is operant as early as 12 days after the first injection in the general population (*72*), the follow-up started at the date of the second dose of vaccine. The Kaplan-Meier method was used to compare COVID-19 incidence in the two populations. Data were censored at either date of death or October 10, 2021. Furthermore, to ensure the accuracy of the comparison, infected patients who did not develop reinfection before immunization were censored at the time of their first mRNA vaccine injection. Also, because in France a systematic third vaccine dose was proposed to all transplant recipients from April 11^th^ onward, vaccinated patients who did not develop COVID-19 before their third dose of vaccine were censored at the time of the third vaccine injection.

The COVATRHUS cohort (COvid-19 VAccine in Transplant Recipients, Hopitaux Universitaires de Strasbourg) was used to analyze immune mechanisms involved in protection against COVID-19. Twenty-nine patients, naive for SARS-CoV-2 infection, were prospectively recruited from the cohort of kidney transplant recipients of the University Hospital of Strasbourg. According to the recommendations of the French health authority, they received two doses of mRNA-1273 (Moderna) SARS-CoV-2 vaccine. A third vaccine injection of mRNA-1273 SARS-CoV-2 vaccine was offered to all patients who did not develop viral neutralizing IgG after the second dose.

Vaccinated patients were compared to 21 patients retrospectively recruited among adult kidney transplant recipients of the University Hospital of Strasbourg, who were diagnosed with COVID-19 between November 1, 2020 and January 31, 2021. The diagnosis of COVID-19 was based on positive testing of nasopharyngeal swabs by reverse transcription-polymerase chain reaction (RT-PCR). The study protocol complied with the tenets of the Helsinki Declaration and was approved by the Institutional Review Board (approval number: 18/21 03, Comité de Protection des Personnes Ouest IV Nantes) and registered on clinicaltrial.gov as NCT04757883. Clinical, demographic, and laboratory data were collected at the time of the first vaccine injection or at the time of the COVID-19 diagnosis. Severity of COVID-19 was graded as asymptomatic, mild, moderate, severe, critical or death following the first WHO recommendations dated May 27, 2020. The immune response after vaccination or infection was assessed at day 14 after the second dose of vaccine or one month after symptoms onset, respectively.

An external validation cohort consisted of non-responders to two doses of BNT162b2 vaccine (Pfizer-BioNtech). These individuals were part of a cohort of kidney transplant recipients of Lyon University Hospital, France. The study protocol was approved by the local Institutional Review Board (approval number: 2020-A02918-31).

### Assessment of cellular immune responses directed against SARS-CoV-2

Peripheral Blood Mononuclear Cells (PBMCs) were collected and isolated by centrifugation on a Ficoll density gradient. The cells were then frozen in fetal calf serum supplemented with 10% dimethyl sulfoxide (DMSO, Sigma-Aldrich). SARS-CoV-2 specific CD8^+^ T cells and CD4^+^ T cells were identified as previously described (*9*, *28*). Briefly, after thawing, cells were concentrated at 10^7^ cells/mL in complete medium (RPMI-1640 Glutamax medium (Invitrogen) supplemented with 10% fetal calf serum, 25 mM Hepes (Invitrogen), and 10 units/mL penicillin/streptomycin (Invitrogen)) and left to rest overnight at 37°C and 5% CO2 in a 96-well round-bottom plate at 10^6^ cells per well. The next day, the RPMI-1640 medium was changed, and the cells were cultured for 24 hours in the presence of peptide pools derived from the viral spike, nucleocapsid and membrane proteins (PepMix, JPT Peptides Technologies GmbH). The pools contained overlapping peptides covering the entire sequence of the indicated viral protein antigens. The final concentration of the peptides was 1μg/mL. Cells cultured with DMSO (Sigma-Aldrich) alone (1:250) were used as negative controls. Cells were then rinsed and incubated at room temperature with the relevant fluorescent antibodies for 30 min: CD3 (UHCT1, Brilliant Violet 421, dilution 1:80, BD Biosciences Cat# 562426, RRID:AB_11152082), CD8 (SK1, allophycocyanin (APC)-H7, dilution 1:80, BD Biosciences Cat# 560179, RRID:AB_1645481), CXCR3 (1C6, Alexa Fluor 488, dilution 1:10, BD Biosciences Cat# 558047, RRID:AB_397008), CXCR5 (RF8B2, Alexa Fluor 647, dilution 1:80, BD Biosciences Cat# 558113, RRID:AB_2737606), CCR6 (11A9, phycoerythrin (PE)-Cyanin (Cy) 7, dilution 1:80, BD Biosciences Cat# 560620, RRID:AB_1727440), CD25 (2A3, PE, dilution 1:50, BD Biosciences Cat# 341011, RRID:AB_2783790), CD4 (SK3, peridinin-chlorophyll-protein (PerCP)-Cy5.5, dilution 1:20, BD Biosciences Cat# 332772, RRID:AB_2868621 or Alexa Fluor 488, dilution 1:10, BioLegend Cat# 344604, RRID:AB_1937227), CD69 (FN50, PE/Dazzle 594, dilution 1:150, BioLegend Cat# 310942, RRID:AB_2564277), CD137 (4B4-1, Alexa Fluor 647, dilution 1:20, BioLegend Cat# 309824, RRID:AB_2566258), and a Fixable Viability Dye (eBioscience, eFluor 510, dilution 1:500). Cells were fixed with 2% methanol-free formaldehyde.

For IFN-γ staining, surface antigen-stained cells were incubated 30 min at 4°C in Fixation/Permeabilization buffer (Foxp3/Transcription Factor Staining Buffer Set from eBioscience). The cells were then rinsed and incubated with anti-IFN-γ fluorescent antibody (4S.B3, PE, dilution 1:10, BD Biosciences Cat# 554552, RRID:AB_395474) in the Permeabilization buffer according to manufacturer instructions. Of note these experiments were performed without Brefeldin A. Samples were acquired on a BD LSR Fortessa 4L flow cytometer (BD Biosciences).

### Assessment of humoral immune responses directed against SARS-CoV-2

IgG directed against the Receptor Binding Domain (anti-RBD IgG) of the spike glycoprotein of the SARS-CoV-2 were detected by a chemiluminescence technique, using the Maglumi SARS-CoV-2 S-RBD IgG test (Snibe Diagnostic) on a Maglumi 2000 analyzer (Snibe Diagnostic), according to the manufacturer’s instructions. This test displays clinical sensitivity and specificity of 100% and 99.6%, respectively. Following WHO recommendation (*73*), titers are expressed as binding antibody units/mL (BAU/mL); the correction factor for Maglumi was 4.33.

The Abbott anti-nucleocapsid (N) IgG assay is an automated chemiluminescence microparticle immunoassay (CMIA) conducted and interpreted according to manufacturer guidelines. A sample-to-calibrator relative light unit index of ≥1.4 is considered positive, an index of ≥0.49 to <1.40 is considered borderline, and an index of <0.49 is considered negative. This CMIA displays clinical sensitivity and specificity of 96.5% and 99.2%, respectively (*74*).

Neutralization assays was performed as follows: 3x10^4^ 293T-ACE2 (provided by O. Schwartz Laboratory, Institut Pasteur) were plated in 96-well plates. Serum samples were sequentially diluted and incubated with D614G spike-pseudotyped lentiviral particles (provided by Rossolillo Laboratory, IGBMC) for 1 hour at 37°C. The mixes were then added to cells. After 72 hours, the intracellular luciferase signal was measured with Bright Glo luciferase assay system by a luminescence Counter MicroBetaTriLux 1450LSC (Perkin Elmer). The percentage of neutralization was calculated as: 100 × (1-(mean(luciferase signal in sample duplicate))/(mean(luciferase signal in virus alone))). The results are reported as the log_10_ of the dilutions that inhibit 50% of the infection of the targets [log_10_(IC50)].

SARS-CoV-2 RBD-specific B cells were identified as previously reported (*75*). Briefly, biotinylated recombinant RBD domain of SARS-CoV-2 RBD (Miltenyi Biotech) was tetramerized either with streptavidin-PE (BD Biosciences) or with streptavidin-APC (BioLegend). Cryopreserved PBMCs were centrifuged and suspended in PEB Buffer (phosphate buffered saline [PBS] plus 0.5% bovine serum albumin [BSA] and 2 mM EDTA) and incubated with Fc receptor block (Miltenyi Biotech) for 15 min at 4 °C (dilution 1:10). Next, cells were washed in PEB and stained for 30 min in brilliant stain buffer at 4 °C in the dark using the following antibodies: anti-CD3 (clone SK7, APC-Fire810, dilution 1:25, BioLegend Cat# 344858, RRID:AB_2860895), anti-CD11c (clone 3.9, Brilliant Violet 785, dilution 1:20, BioLegend Cat# 301644, RRID:AB_2565779), anti-IgD (clone IA6-2, Brilliant Violet 605, dilution 1:50, BioLegend Cat# 348232, RRID:AB_2563337), anti-CD19 (clone LT19, PE-Vio770, dilution 1:50, Miltenyi Biotec Cat# 130-113-170, RRID:AB_2733209), anti-CD27 (clone M-T271, PerCP-Vio700, dilution 1:50, Miltenyi Biotec Cat# 130-113-632, RRID:AB_2784096), anti-CD38 (clone REA572, VioBright fluorescein isothiocyanate (FITC), 1:25, Miltenyi Biotec Cat# 130-113-433, RRID:AB_2726165), anti-CD20 (clone 2H7, Brilliant Violet 421, dilution 1:25, BD Biosciences Cat# 562873, RRID:AB_2737857), anti-CD21 (clone B-ly4, Brilliant Ultra-Violet 496, dilution 1:100, BD Biosciences Cat# 750614, RRID:AB_2874746), together with both PE- and APC-conjugated recombinant RBD tetramers. Cells were washed in PEB and resuspended in a PEB dilution (1:500) of the fixable viability dye eFluor 780 (eBioscience, eFluor 780, dilution 1:500). They were next washed and fixed at 4% paraformaldehyde (PFA) for 20 min at 4 °C in the dark before a final wash and resuspension for analysis. Samples were then acquired on a Cytek Aurora spectral flow cytometer equipped with five lasers operating on 355nm, 405nm, 488nm, 561nm and 640nm using the SpectroFlo V2.2.0 (Cytek) software. Data were analyzed using FlowJo10.6.1 software (Becton Dickinson). Because our interest was on the ongoing humoral immune response (antigen-experienced B cells), we excluded from analysis naïve B cells (CD19^+^ CD20^+^ IgD^+^ CD27^-^).

### Statistical analysis

Raw, individual-level data for experiments where n<20 are presented in data file S1. All the analyses were carried out using GraphPad Prism v8.0. Qualitative variables were expressed as percentages and compared with the chi-square test or Fisher’s exact test when the conditions of application of chi-square were not met. Due to lack of normal distribution of some variables in the epidemiological cohort or small sample size in the mechanistic cohort, quantitative variables were all expressed as median ± interquartile range (IQR) and compared using Mann-Whitney test. Paired data were compared using Wilcoxon test. All tests were two-sided. Incidence data was analyzed by Kaplan-Meier plot and were compared using a Log-rank test. Non-linear regression was performed to study the correlation of continuous quantitative variables.

## Supplementary Materials

### Other Supplementary Material for this manuscript includes the following:

MDAR Reproducibility Checklist

## List of supplementary materials

Figure S1 Table S1 MDAR Reproducibility Checklist Data file S1
