# Supplementary material for: Infection or a third dose of mRNA vaccine elicits neutralizing antibody responses against SARS-CoV-2 in kidney transplant recipients
Source: Sci Transl Med. 2022 Feb 1:eabl6141. doi: 10.1126/scitranslmed.abl6141 (PMC8939774; doi:10.1126/scitranslmed.abl6141)
Supplement: Supplementary file 1 — Fig. S1 Table S1 [file scitranslmed.abl6141_sm.pdf]

Science Translational Medicine
AAAS

Supplementary Materials for

Infection or a third dose of mRNA vaccine elicit neutralizing antibody responses against SARS-CoV-2 in kidney transplant recipients

Xavier Charmetant *et al.*

Corresponding author: Olivier Thaunat, olivier.thaunat@chu-lyon.fr

DOI: 10.1126/scitranslmed.abl6141

The PDF file includes:

Fig. S1
Table S1

Other Supplementary Material for this manuscript includes the following:

MDAR Reproducibility Checklist
Data file S1

Supplementary materials

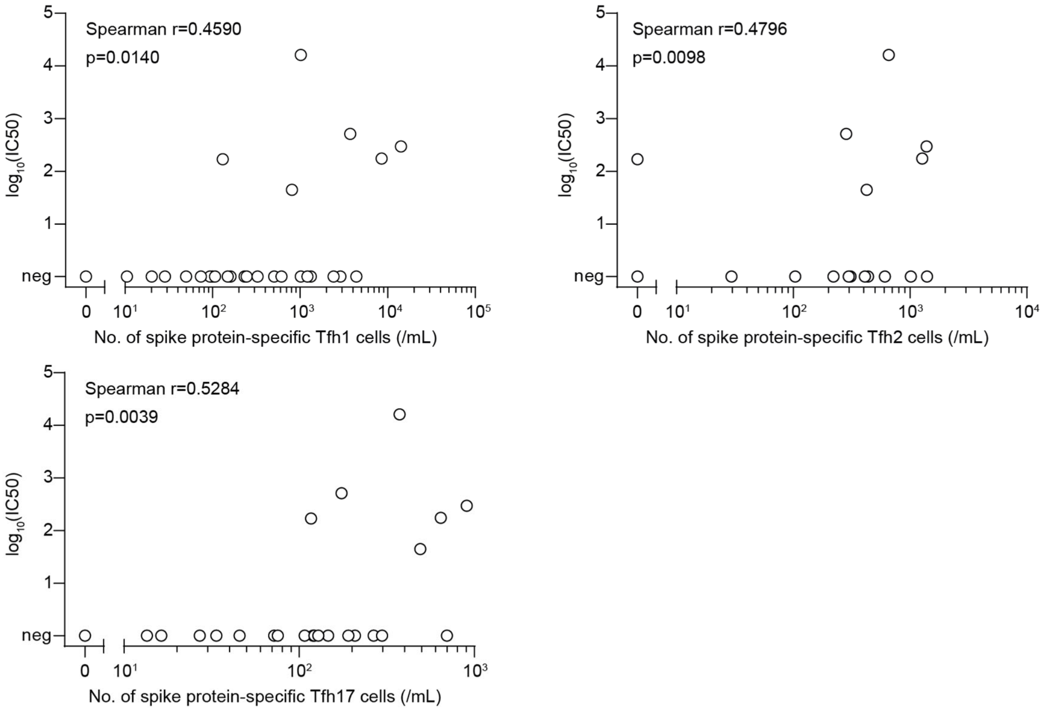

Fig. S1. Correlations between Tfh subpopulations and the viral neutralization capacity of the serum. The correlations between the number of spike protein-specific Tfh1, Tfh2, or Tfh17 cells and the viral neutralization capacity of the serum are shown. The results of Spearman correlation test are shown on the graphs. Tfh, T follicular helper. Neg indicates absence of neutralizing antibodies.

Table S1. Clinical characteristics of epidemiological and COVATRHUS cohorts.

n (%) or median [IQR]	Epidemiological cohort Infected n= 137	Epidemiological cohort Vaccinated n= 736	p[1]	COVATRHUS cohort Infected n=21	COVATRHUS cohort Vaccinated n=29	p[2]	Inf p[3]	Vac p[4]
**Age (y)**	59.9 [49.7-68.9]	57.7 [49.1-67.3]	0.219	54.9 [36.9-60.4]	56.4 [43.4-69.6]	0.314	0.057	0.819
**Male**	88 (64)	439 (60)	0.361	12 (57)	18 (62)	0.726	0.530	0.794
**BMI (kg/m²)**	26.0 [23.0-31.0]	26.0 [22.6-30.0]	0.404	25.0 [22.5-30.0]	24.2 [23.4-27.5]	0.603	0.543	0.259
**Time since transplantation (y)**	6.18 [2.14-12.6]	6.41 [2.94-13.1]	0.176	2.0 [1.35-7.53]	7.04 [2.19-16.2]	**0.017**	**0.027**	0.846
**Donor type**			0.652			0.08	0.140	0.544
Deceased	106 (77)	602 (82)		13 (62)	25 (86)			
Living	27 (20)	134 (18)		7 (33)	4 (14)			
NA	4 (3)	0		1 (5)	0			
**Comorbidities**								
Cardiovascular disease	48 (35)	226 (31)	0.367	4 (19)	21 (72)	**<0.001**	0.146	**<0.001**
Diabetes	58 (42)	263 (36)	0.169	7 (33)	3 (10)	**0.045**	0.435	**0.005**
**Induction**			**<0.001**			0.087	0.797	0.743
Anti-thymocyte globulins	47 (34)	418 (57)		8 (38)	17 (59)			
Basiliximab	80 (58)	254 (34)		12 (57)	9 (31)			
No induction	7 (5)	38 (5)		0	0			
NA	3 (2)	26 (4)		1 (5)	1 (3)			
**Immunosuppressive drugs**								
CNI	126 (92)	659 (90)	0.385	19 (90)	28 (97)	0.372	0.817	0.221
MMF/MPA	109 (80)	582 (80)	0.898	19 (90)	22 (76)	0.184	0.235	0.677
Steroids	89 (65)	493 (67)	0.645	14 (67)	19 (66)	0.932	0.879	0.869
imTOR	22 (16)	126 (17)	0.751	1 (5)	3 (10)	0.472	0.172	0.336
Belatacept	3 (2)	24 (3)	0.506	2 (10)	1 (3)	0.372	0.074	0.956
Imurel	3 (2)	16 (2)	0.991	0	0	NA	NA	NA

Qualitative variables were compared using a Chi-square test, quantitative variables were compared using a Mann Whitney test. Significant p-values are in bold. p[1] was the comparison between infected (Inf) and vaccinated (Vac) patients from the epidemiological

cohort; p^2 was the comparison between infected and vaccinated patients from the biological cohort; p^3 was the comparison between infected patients from the epidemiological and biological cohorts; p^4 was the comparison between vaccinated patients from the epidemiological and biological cohorts. IQR, interquartile range; BMI, body mass index; SD, standard deviation; y, years; NA, not available; CNI, calcineurin inhibitor; MMF/MPA, mycophenolate mofetil/mycophenolic acid; imTOR, inhibitors of the mechanistic target of rapamycin.
